# Development and validation of a web-based questionnaire to identify environmental risk factors for inflammatory bowel disease: the Groningen IBD Environmental Questionnaire (GIEQ)

**DOI:** 10.1007/s00535-018-1501-z

**Published:** 2018-08-14

**Authors:** Kimberley Wilhelmina Johanna van der Sloot, Rinse Karel Weersma, Gerard Dijkstra, Behrooz Zaid Alizadeh

**Affiliations:** 1 Department of Gastroenterology and Hepatology, University of Groningen and University Medical Center Groningen, PO Box 30.001, 9700RB Groningen, The Netherlands; 2 Department of Epidemiology, University of Groningen and University Medical Center Groningen, Groningen, The Netherlands

**Keywords:** Exposome, Environmental factors, Inflammatory bowel diseases, Questionnaire, Validation

## Abstract

**Background:**

In the complex etiology of inflammatory bowel disease (IBD), the exposome is a major contributor. Though many environmental exposures have been identified, quality of evidence varies greatly and overall evidence for the exposome is inconclusive. A universal, precise, and reproducible measurement tool is needed to study the exposome in IBD.

**Methods:**

We built the web-based Groningen IBD Environmental Questionnaire (GIEQ), an extensive and structured questionnaire measuring potentially involved environmental exposures, consisting of 848 items, subdivided into 15 categories. For validation, 76 IBD patients completed the GIEQ twice (2-month interval). Cohen’s kappa and correlation coefficients were used to compare both fills. Internal consistency was evaluated using Cronbach’s alpha tests. Proportional bias was examined using Bland–Altman plots.

**Results:**

In general, we obtained a mean kappa coefficient of 0.78 (standard deviation 0.17) for categorical questions and a mean intraclass correlation coefficient of 0.88 (0.15) for numeric questions. Cronbach’s alpha ranged from 0.64 to 1.0 with a mean of 0.79 (0.14). Bland–Altman plots showed proportional bias only for current physical activity score.

**Conclusions:**

The GIEQ is a reliable measurement tool to study the exposome in IBD, enabling consistent measurement of an extended number of environmental factors and their interactions. Use of the GIEQ across IBD cohorts will lead to more standardized, generalizable, and comparable results. Also, the GIEQ can be used for calculation of an exposome risk score, applicable for secondary prevention by identifying high-risk patients as well as to analyze interactions between the exposome and other aspects of IBD etiology.

**Electronic supplementary material:**

The online version of this article (10.1007/s00535-018-1501-z) contains supplementary material, which is available to authorized users.

## Introduction

Inflammatory bowel disease (IBD), consisting of ulcerative colitis (UC) and Crohn’s disease (CD), is a gastrointestinal disease characterized by chronic inflammation [1]. Disease etiology is complex; besides the clear role of genetic susceptibility, increasing evidence indicates an important role for lifestyle and environment [2–5]. Originally, IBD was a western lifestyle-mediated disease, with incidence rates highest in Europe (37.0 per 100,000 person–years) and the United States (39.4 per 100,000 person–years). However, with global westernization and changing lifestyles, incidence rates of IBD are now rising in developing countries as well, making IBD a global health problem [6].

Previously, we presented a comprehensive overview of the current state of knowledge concerning proposed environmental factors forming the exposome [7]. Numerous environmental exposures, starting at birth, have been associated with the development of IBD in past studies [4, 7–9]. Different markers of childhood hygiene, in line with the hygiene hypothesis, as well as receiving breastfeeding are shown to be protective against IBD, whereas antibiotic use during childhood increases risk of CD alone [10–14]. Later in life, other environmental exposures come into play, e.g., the use of hormone-containing medications and non-steroidal anti-inflammatory drugs (NSAIDs), increasing chances of developing IBD [15–17]. Cigarette smoking, on the other hand, holds a divergent effect, as a protective effect is described for UC, while risk of CD development increases [18]. Physical activity might protect against CD, but evidence is incoherent [19]. Also, exposures concerning living environment seem to play a role in disease development regardless of time of exposure, such as living in southern latitudes as shown by a Scandinavian study, a high summer temperature, and an increased concentration of (predicted) vitamin D, all thought to play a protective role against development of IBD, opposite to the potential role of air pollution [20–22].

Although exploring of all these environmental exposures has led to new steps in understanding disease etiology, this knowledge was not translated to recommendations, and clinical applicability has not been practiced due to a number of limitations. First of all, the quality of evidence varies greatly among different factors [7]. Whereas the protective role of breastfeeding has been shown in a comprehensive meta-analysis, the association of recently identified factors such as air pollution with IBD has only been shown in single-center case-controlled or cohort studies, and replication of results is lacking [22, 23]. Second, different environmental factors are often studied in different patient cohorts. Consequently, possible interactions within the exposome remain unexamined. Finally, the role of environmental exposures and the exposome is often studied without taking the importance of genetic susceptibility into account. Hypothesizing that genetic susceptibility forms the starting point of disease development, each environmental exposure involved in disease etiology, starting at birth, forms an additional hit in the complex process of IBD development.

**Fig. 1:**
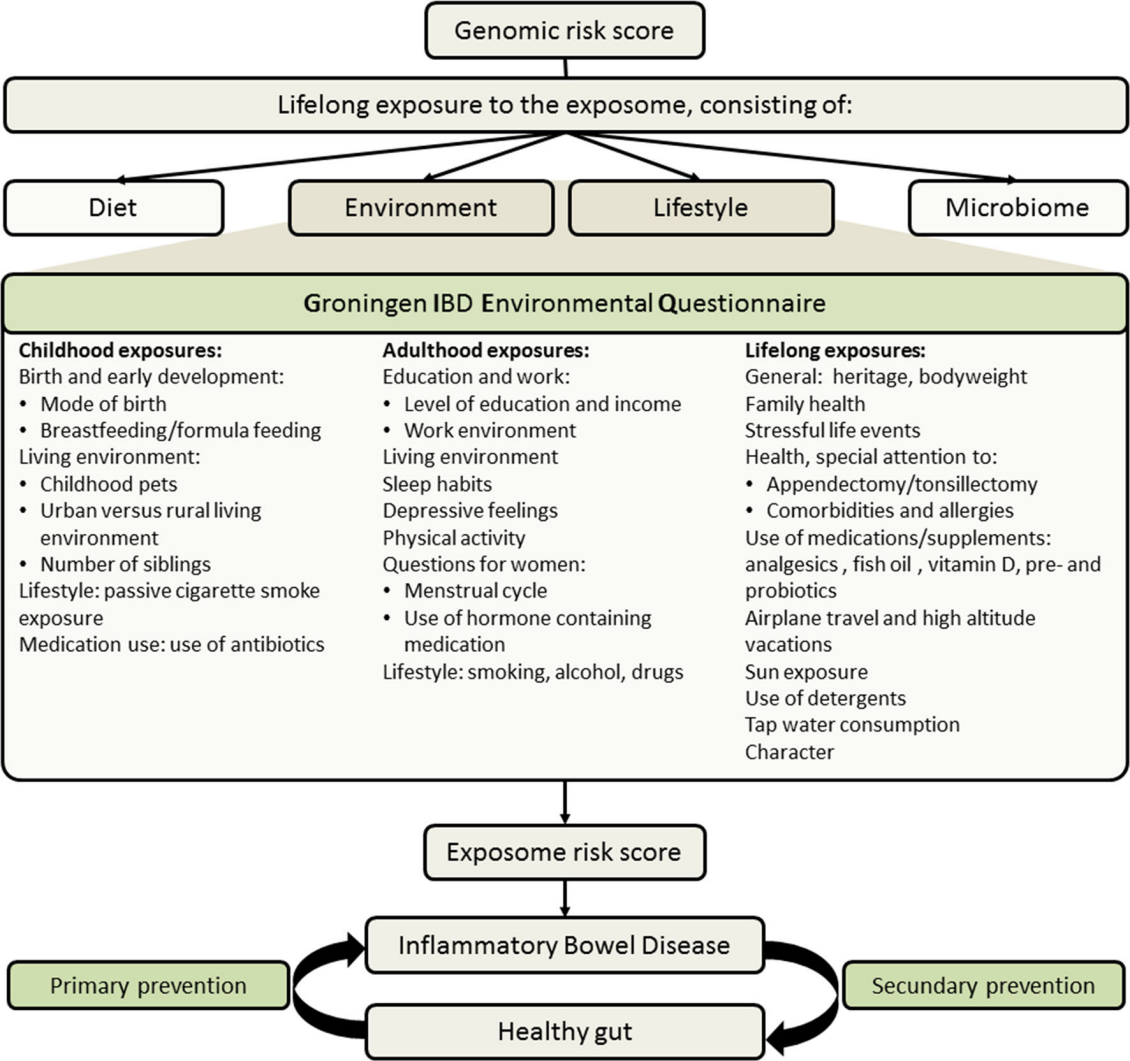
Role of the Groningen IBD Environmental Questionnaire in disease etiology

Since not all identical twins, as well as not all individuals with equal environmental exposures will continue to develop IBD, interactions between the genome and exposome, therefore, have to play a crucial role in disease development. Whereas knowledge concerning the role of the genome in disease etiology has improved greatly in the past years, the exposome has fallen behind [2, 4]. Together with the microbiome and diet, the exposome and genome form the basis of the complex model of IBD etiology (Fig. 1). Given the lack of consistent high quality evidence for the role of environmental factors for IBD, subsequent steps have to be taken to fill these gaps in the understanding of disease etiology and to provide sufficient and convincing evidence supporting the role of the exposome in IBD and thus for its clinical applicability. Possibly involved environmental exposures forming the exposome should comprehensively be measured in large, well-documented study populations using a validated and universally applicable tool. Ultimately, an exposome risk score (ERS) could be build, following in the footsteps of the genetic risk score (GRS) [2, 24].

Whereas measurement methods of the genome are very consistent and comparable across IBD cohorts, and were standardized and harmonized by consortia, due to using common array technologies and calling methodologies, a comprehensive measurement method for the exposome is lacking. Previous studies have used the environmental factors scheme of the International Organization of Inflammatory Bowel Disease (IOIBD) [8]. However, this questionnaire is inapplicable for studying a westernized population due to i. subject selection (i.e., sanitary conditions and childhood vaccinations), as there will be little to no differences across these examined factors within patient cohorts, and ii. the fact that several (recently) described factors are not included in this questionnaire. Therefore, to further our understanding of the role of the exposome, the first step consists of the formation of an IBD-specific, reliable, reproducible, and universally applicable measurement tool to examine the exposome across IBD cohorts. Therefore, we have built and validated the Groningen IBD Environmental Questionnaire, hereafter referred to as the GIEQ, evaluating a comprehensive of possibly involved exposures. Combining both entities of ERS and GRS will possibly lead to the identification of individuals at risk for IBD, an earlier diagnosis when symptoms occur in high-risk individuals, development of recommendations for lifestyle in at risk individuals, and contribution to a more personalized management plan of disease [25].

## Methods and materials

**Fig. 2:**
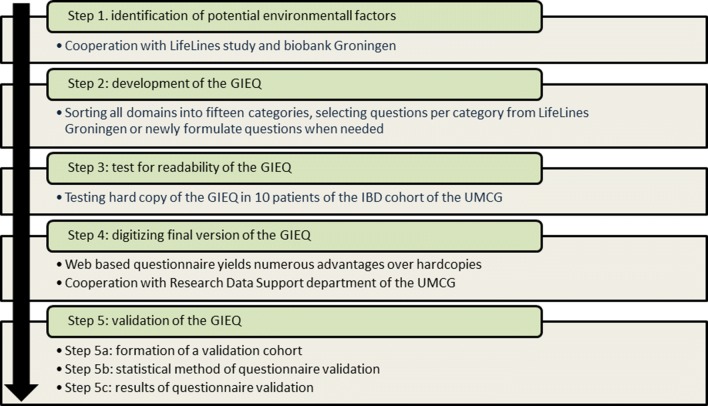
Flowchart of development of the GIEQ

To build a reliable and reproducible measurement tool to study the exposome in IBD, a number of consecutive steps have been followed, which will be discussed in chronological order (Fig. 2).

### Step 1: Identification of potential environmental factors

In building the GIEQ, we cooperated with the LifeLines study and biobank in Groningen, the Netherlands. LifeLines is a multi-disciplinary prospective population-based cohort study examining in a unique three-generation design, the health and health-related behaviors of 167,729 persons living in the North of the Netherlands. It employs a broad range of investigative procedures in assessing the biomedical, socio-demographic, behavioral, physical, and psychological factors which contribute to the health and disease of the general population, with a special focus on multi-morbidity and complex genetics. Questionnaire domains were selected, based on current knowledge of already known environmental factors, as summarized in our review, and possibly involved, novel environmental factors, allowing us to study and discover yet unknown environmental risk factors involved in IBD [7, 26]. An overview of selected domains, ordered over time, is shown in Fig. 1.

### Step 2: Development and content of the GIEQ

Next, all domains were sorted into 15 categories. Questions examining these domains were either selected from the previously validated questionnaires used in the LifeLines cohort and biobank study, or newly formulated by us [26]. Questions were either categorical, numeric, or narrative text, and ordinal answering categories were used when applicable. Often, questions are asked for two distinct time points, once to examine the exposure before disease diagnosis, once for the time at study recruitment of the patient. Overall, the GIEQ consists of 848 items (587 categorical, 228 numeric, and 33 narrative items), spread over 15 categories: general (18 items), birth and development (13), family health (141), work and education (21), living environment (47), sleep (48), physical activity (114), stress (52), health (78), allergy (24), medication use (37), questions for females (16), lifestyle (110), detergents (28), and character (101). Examples of questions are further described in the results section and Online supplementary material—the GIEQ.

The questionnaire was then evaluated for meeting its aim, its content, structure consistency, and persistency, and its contextual coherence. All individual questions including answering categories and source are presented in the online supplementary material—the GIEQ.

### Step 3: Readability of the GIEQ (pilot study)

To test for readability, duration, and clarity of the GIEQ for IBD patients, a hardcopy of the GIEQ was distributed among ten IBD patients from the specialized IBD-Center at the University Medical Center Groningen, the Netherlands. Patients were selected at random from the 1000IBD cohort of the UMCG, and were asked to fill the GIEQ with special attention to usability, difficulty in reading, incomprehensiveness of questions, options offered for each question, and time spent to complete questionnaire. Whereas overall evaluation of the questionnaire was positive and comprehensive, taking approximately 60 min to be completed, patients mainly commented on the lack of appropriate answering categories for a number of questions. All comments were evaluated and recommendations were accommodated accordingly to specified questions.

### Step 4: Digitizing the GIEQ

A web-based questionnaire offers patients a more convenient way of participating in a questionnaire-based study, accompanied by a decreased chance of filling errors, due to the smart web application design. Wrongful answers are minimized by only unfolding follow-up questions when appropriate based on previous answers. When a patient indicates that a certain exposure is not applicable to him or her, follow-up questions will not appear. For numeric questions, clear range borders are set, to prevent impossible answers. Together, a web-based version of the GIEQ offers a more reliable method of data collection and minimizing possible subjective errors and also decreasing the overall duration of filling the questionnaire, hindering the source of biases. Therefore, the GIEQ was digitized in close cooperation with experts in developing online tools at the Research Data Support (RDS) unit at UMCG. Strict steps have been undertaken to keep privacy of patients secured based on ethical and scientific integrity guidelines of the UMCG. After digitizing the GIEQ, questions were checked again for their accuracy.

### Step 5: Validation of the GIEQ

Once formation of the GIEQ was completed from steps 1–4, several steps have been undertaken to test for reliability and reproducibility.

### Step 5a: Formation of a validation cohort

The first 300 patients of the 1000IBD cohort at the UMCG were invited to participate in the GIEQ-study. This IBD cohort consists of patients treated in the IBD-Center of the Department of Gastroenterology and Hepatology of the UMCG for whom extensive multi-omics information has been collected including, among others, genome, transcriptome, microbiome, and dietary information. Patients are prospectively followed, and extensive information on disease diagnosis and course is collected during routine visits to the IBD center. (Imhann et al., submitted) Patients were invited to participate by letter or phone call. Approximately 70% of invited patients initially enrolled, after which 148 patients completed the GIEQ. These 148 patients were all asked to fill the GIEQ a second time, approximately 2 months after receiving their first filled GIEQ, after which a validation cohort was formed (N:76). Due to the possibility of selection bias, baseline characteristics and the Montreal classification (up to time of survey) of the validation cohort were compared to the remaining IBD cohort, using chi-square tests for categorical variables and Kruskal–Wallis *H* tests for continuous variables. The results are shown in Table 1. Compared to the complete IBD cohort, patients of the validation cohort were statistically significantly older, accompanied by a longer mean disease duration. These differences are not likely to influence questionnaire validation results.

**Table 1:** Baseline characteristics of the validation cohort in comparison to the 1000IBD cohort

		IBD cohort	Validation cohort
		*N*: 1341	*N*: 76
Age^a^	Mean ± SD	45.7 (15.7)	51.5 (13.2)
Sex			
Male	*n* (%)	562 (41.9)	35 (46.1)
Female	*n* (%)	779 (58.1)	41 (53.9)
IBD type			
Crohn’s disease	*n* (%)	699 (52.1)	38 (50.0)
Ulcerative colitis	*n* (%)	570 (42.5)	36 (47.4)
IBD unclassified	*n* (%)	72 (5.4)	2 (2.6)
Disease duration^a^	Mean ± SD	15.8 (10.2)	19.1 (11.0)
History of cigarette smoking	*n* (%)	733 (54.8)	42 (57.5)
Montreal classification			
Age at diagnosis			
< 16 years	*n* (%)	191 (14.2)	9 (11.8)
17–40 years	*n* (%)	847 (63.2)	44 (57.9)
> 40 years	*n* (%)	299 (22.3)	23 (30.3)
Disease location (CD)			
Ileal disease	*n* (%)	249 (35.6)	11 (28.9)
Colonic disease	*n* (%)	144 (20.6)	9 (23.7)
Ileocolonic disease	*n* (%)	287 (41.1)	18 (47.4)
(Isolated) upper GI-disease	*n* (%)	71 (10.2)	3 (7.9)
Disease behavior (CD)			
Inflammatory	*n* (%)	342 (48.9)	13 (34.2)
Structuring	*n* (%)	231 (33.0)	14 (36.8)
Penetrating	*n* (%)	111 (15.9)	11 (28.9)
Perianal disease	*n* (%)	207 (30.3)	12 (31.6)
Disease extent (UC)			
Proctitis	*n* (%)	58 (10.2)	3 (8.3)
Left sided UC	*n* (%)	167 (29.3)	16 (44.4)
Extensive UC	*n* (%)	300 (52.6)	14 (38.9)
Disease severity (UC)			
Asymptomatic	*n* (%)	36 (6.3)	1 (2.8)
Mild UC	*n* (%)	151 (26.5)	13 (36.1)
Moderate UC	*n* (%)	187 (32.8)	12 (33.3)
Severe UC	*n* (%)	137 (24.1)	6 (16.7)
Complicated disease course			
Need for biologicals	*n* (%)	492 (36.8)	20 (26.7)
Need for surgery	*n* (%)	442 (33.0)	32 (42.1)

^a^Indicates a level of significance < 0.01

SD indicates standard deviation, number and (percentages) as *n* indicated *n* (%)

### Step 5b: Questionnaire validation

Next, all individual questions forming the GIEQ were evaluated for reliability and reproducibility by comparing the first fill (Q1) to the second fill 2 months later (Q2), for each of the 76 individuals. Descriptives and distribution of each question was checked. Clear outliers and impossible answers (e.g., working 8 days per week) were excluded from further analysis. For categorical questions, Cohen’s kappa coefficients were calculated to determine level of agreement between Q1 and Q2. As standard practice, questions scoring a kappa coefficient above 0.6 were deemed valid [27–29]. In categorical questions with five or more answer possibilities, answering categories plus and minus one category were deemed equal. For continuous questions, either Pearson or Spearman correlation coefficients, based on variable distribution, were applied to correlate questions answers between Q1 and Q2 per each question. A cutoff value of 0.6 was set also, above which questions were deemed valid. In addition, the Intraclass Correlation Coefficients (ICC) were calculated for continuous variables. When correlation was low, Bland–Altman plots were used to examine the possibility of proportional bias [30]. Independent questions with low reliability were removed from further analysis and the GIEQ, questions which were part of a series of questions of a given domain (for example daily physical activity) and had a low individual reliability could not be excluded. Mann–Whitney *U* tests were used to examine possible differences in reliability when items were used twice, to assess time before disease diagnosis and the time at interview. To determine internal consistency of the GIEQ, Cronbach’s alpha was determined if possible, and a mean was calculated per category. Statistical tests chosen for each question of the GIEQ individually and its results are presented in the online supplementary material—the GIEQ. Finally, to further analyze the consistency of responses between the GIEQ and data collection by patient interviews by treating physicians, we compared a subset of results of the GIEQ to available matching data, separately collected in the longitudinal and prospective 1000IBD cohort, using kappa and Spearman correlation coefficients.

## Results

**Fig. 3:**
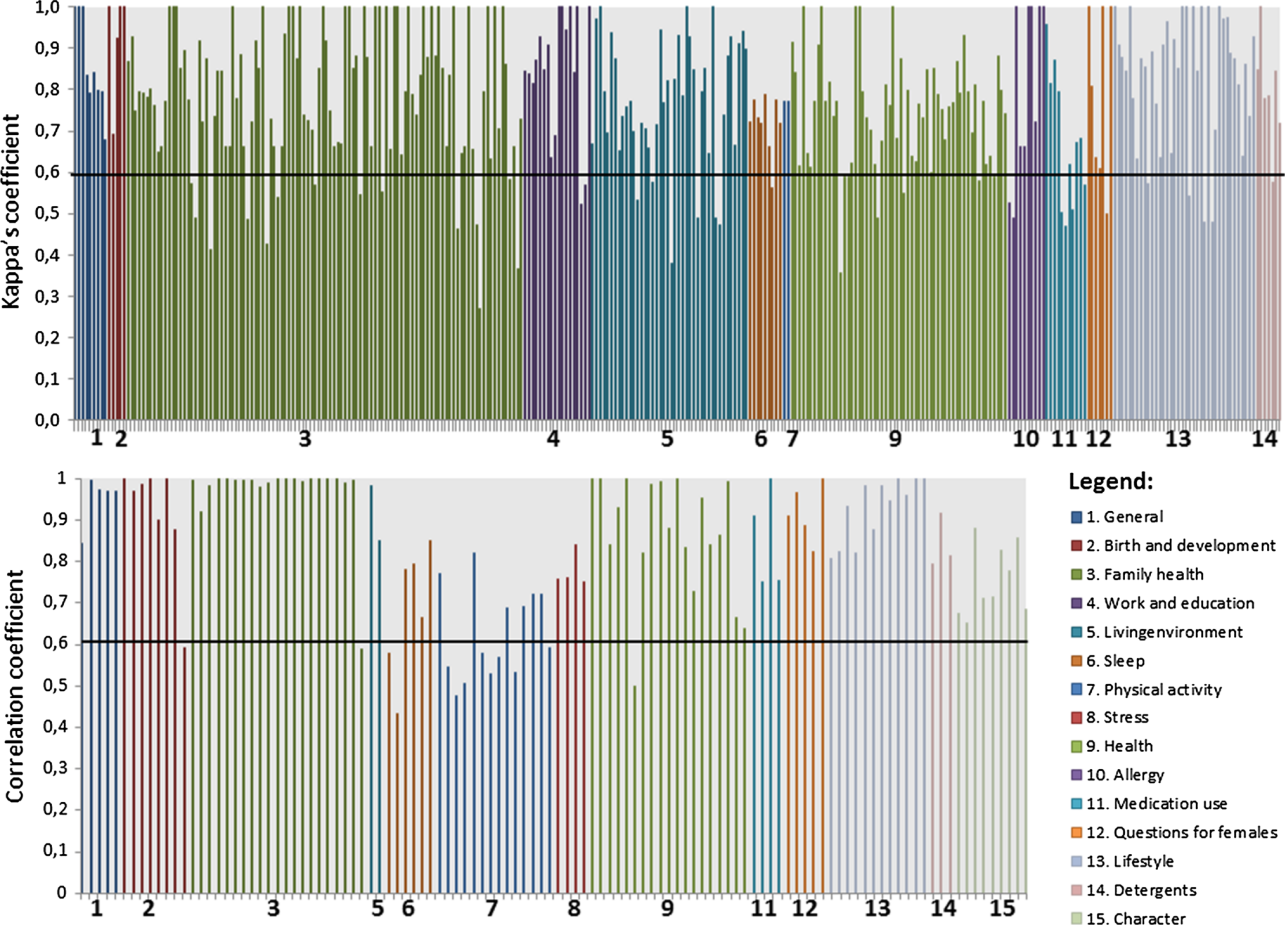
Reliability of individual GIEQ items, stratified by category

Overall, a mean Cohen’s kappa coefficient of 0.78 for all 584 categorical items (standard deviation 0.17) was achieved, indicating a substantial level of agreement. However, large variation exists between questionnaire categories, ranging from 0.68 (0.16) for medication use indicating moderate agreement to 0.92 (0.13) for items concerning birth and development, indicating almost perfect agreement. A mean overall correlation coefficient of 0.85 (0.16) was found for all 215 numeric items, ranging from a mean of 0.62 (0.11) of items concerning physical activity, to 1.00 (0.00) when family health involving ones’ children is reviewed. Figure 3 displays an overview of either Cohen’s kappa coefficient or correlation coefficients of each individual item, sorted per category. An overview of validation statistics per category is shown in Table 2. Due to the differences between categories, all will be discussed separately below.

**Table 2:** Validation data per each of the 15 categories constituting the GIEQ

	Kappa (SD)	*R* (SD)	ICC (SD)	Cronbach’s α	No. of items^a^
General	0.86 (0.12)	0.95 (0.06)	0.95 (0.09)		14
Birth and development	0.92 (0.13)	0.92 (0.14)	0.93 (0.14)		13
Family	0.79 (0.17)	0.97 (0.09)	0.98 (0.04)	0.92	128
General	0.82 (0.07)	0.97 (0.04)	0.98 (0.02)		10
Parents	0.79 (0.16)	1.00 (0.00)	1.00 (0.00)		62
Children	0.85 (0.16)	0.99 (0.01)	0.99 (0.02)		21
Siblings	0.76 (0.21)	0.95 (0.14)	0.97 (0.07)		35
Work and education	0.85 (0.15)	–	–	0.84	18
Living environment	0.77 (0.16)	0.92 (0.09)	0.95 (0.05)		44
Sleep	0.72 (0.07)	0.68 (0.16)	0.80 (0.12)	0.78	15
Physical activity	0.77 (0.00)	0.62 (0.11)	0.60 (0.14)		16
Stress	–	0.78 (0.04)	0.87 (0.03)	0.64	4
Health	0.71 (0.18)	0.87 (0.15)	0.90 (0.10)	0.78	76
Allergies	0.81 (0.22)	–	–		10
Medication use	0.68 (0.16)	0.85 (0.12)	0.92 (0.08)	0.74	15
Questions for females	0.79 (0.21)	0.92 (0.07)	0.94 (0.06)		12
Lifestyle	0.83 (0.16)	0.93 (0.08)	0.94 (0.05)	1.00	51
Watching television	–	0.82 (0.01)	0.90 (0.01)		2
Smoking	0.83 (0.14)	0.93 (0.07)	0.95 (0.06)		17
Use of alcohol	0.80 (0.14)	–	–		6
Drug use	0.83 (0.21)	0.99 (0.02)	0.97 (0.04)		17
Traveling	0.93 (0.05)	–	–		4
Sun exposure	0.79 (0.11)	–	–		5
Detergents	0.79 (0.13)	0.84 (0.07)	0.91 (0.04)		10
Character^b^	–	0.75 (0.08)	0.87 (0.05)	0.74	9

^a^Based on number of variables included in validation analysis, does not equal number of GIEQ items since often sum scores are used

^b^Based on sum scores calculated from a 64-item character questionnaire

SD indicates standard deviation, ICC indicates intraclass correlation coefficient, R indicates the mean correlation coefficient, either Spearman or Pearson, based on normality of the tested variable, P indicates the level of significance

In the general category, items mainly concern demographic information and, e.g., weight. With a mean Cohen’s kappa coefficient of 0.86 (ranging from 0.68 to 1.00) in categorical variables and a mean correlation of 0.95 (0.84–1.00) for continuous variables, sufficient agreement was obtained.

When focusing on birth and development, items ranging from weight at birth to receiving breastfeeding or formula as an infant, a mean Cohen’s kappa coefficient of 0.88 (0.69–1.00) was gained. Continuous variables showed a high correlation of 0.95 (0.78–1.00).

For items concerning family health, a clear difference was observed when comparing items concerning ones’ children (Cohen’s kappa coefficient of 0.85, 0.55–1.00) to ones siblings (0.70, − 0.10–1.00), although low numbers of (positive) answers might decrease the coefficient dramatically. Therefore, caution is needed when sibling health is evaluated by the GIEQ in further studies.

When evaluating education and work, a mean Cohen’s kappa coefficient of 0.85 (0.52–1.00) was shown. In this category, separate analysis of items concerning the time before disease diagnosis (0.85, range of 0.64–1.0) and the current situation (0.84, 0.52–1.00), shows no difference in agreement as was tested with a Mann–Whitney test (*p* value 0.74).

When addressing living environment, items concerning childhood pets and living surroundings (e.g., rural versus urban), among others, we got a mean agreement of 0.74 (− 0.02–1.00). As mentioned before, in sub questions, a low number of (positive) responses also might decrease the observed Cohen’s kappa coefficient drastically.

For analyzing sleep, a mean agreement of 0.71 (0.56–0.79) and 0.67 (0.46 – 0.85) was observed for categorical and continuous items, respectively. With a mean ICC of 0.80 (0.60–0.92), however, substantial agreement was obtained. When comparing items concerning time before diagnosis (Cohen’s kappa coefficient 0.74, 0.72–0.78, correlation coefficient 0.60, 0.46–0.78) to items evaluating the present (0.70, 0.56–0.79 and 0.77, 0.67–0.85), no significant difference was observed for either categorical items (p value 0.51) nor continuous items (p value 0.25). Bland–Altman plots showed no evidence for proportional bias. Please see online supplementary material 2—Bland–Altman plots, for all individual plots.

Examining physical activity (PA) is previously shown to be difficult [19]. Using the Short Questionnaire to ASses Health (SQUASH) evaluating PA, a mean correlation of 0.62 (0.48–0.82), measured by Spearman coefficients was retained, comparable to the previous results of the SQUASH deemed valid [31]. Since zero activity in highly unlikely, participants with a zero-activity score were excluded from analysis due to the high likelihood of incorrect answering. ICC showed a similar agreement of 0.60 (0.34–0.82). When comparing agreement between applying the SQUASH to time before disease diagnosis (0.60, 0.48–0.82) and at the time of interview (0.64, 0.53–0.72), no significant differences were found (*p* value 0.34). Bland–Almont plots showed proportional bias for total PA score before disease diagnosis (*p* value 0.03), but not on present PA score (*p* value 0.38).

Sum scores evaluating stress as caused by unpleasant life-events showed a mean agreement of 0.78 (0.75–0.84), with no significant difference when comparing time before disease (0.76, 0.76–0.76) to time at interview (0.80, 0.75–0.84, p value 1.00).A mean ICC of 0.87 (0.85–0.91) was observed, with no indication of proportional bias, as shown by Bland–Altman plots.

Items evaluating health primary focus on symptoms of other autoimmune diseases, such as bronchial hypersensitivity, eczema, alopecia and vitiligo. A mean agreement of 0.73 (0.27–1.00) and 0.92 (0.62–1.00) was found for categorical and continuous items, respectively.

Food allergies were analyzed separately, for which a mean agreement for categorical items of 0.84 (0.49–1.00) was found. This category does not contain continuous items.

Medication use assesses the use of over the counter medications, with extra focus on analgesics and food supplements such as fish oil and vitamin D. A low agreement of 0.52 (0.05–0.96) is found when all items are combined. Separate evaluation of items assessing time before (0.60, 0.32–0.96) and after (0.44, 0.05–0.68) diagnosis shows no significant difference (*p* value 0.08), although a trend seems present. Only a subset of items assessing the amount of analgesics in the year before diagnosis shows convincing agreement (0.88, 0.82–0.96). All other items have been excluded from the GIEQ.

Questions for women, concerning the menstrual cycle and the use of hormone-containing medications have shown a good overall agreement for categorical (0.79, 0.5–1.00) and numeric (0.89, 0.72–1.00) questions.

Items evaluating several different aspects of lifestyle, varying from watching television to alcohol and drug use, show a good overall agreement of 0.84 (0.48–1.00) and 0.92 (0.75–1.00) for categorical and continuous items, respectively.

Use of detergents covers exposure to toothpaste and dishwashing soap, among others. Overall agreement of categorical (0.84, 0.48–1.00) as well as continuous items (0.92, 0.75–1.00) was shown to be sufficient.

At last, character was assessed by using the NEO character questionnaire. Whereas agreement of individual questions was low (0.37, 0.02–0.74), when these 64 items were combined in sum scores of eight different personality traits (competence, anger-hostility, self-consciousness, impulsivity, excitement seeking, self-discipline, vulnerability, and deliberation), agreement increased greatly (0.77, 0.67–0.88). Supplementary Fig. 1 provides the reliability of individual items per category.

Supplementary Table 1 displays comparison of the outcomes as measured by the GIEQ to comparable, prospectively collected data in the 1000IBD cohort. With a mean reliability of 0.89 (0.78–1.0), almost perfect agreement is shown.

## Discussion

We present a validated, universal measurement tool to evaluate the exposome in patients with IBD. Whilst some categories have proven to be more reliable than others, overall reliability and reproducibility are substantial. Level of reliability of each individual question and attributing category can to be taken into account when interpreting future results of the GIEQ. Altogether, the GIEQ is shown to be a reliable tool to measure environmental exposures in IBD, is readily available to the research community, and can be used to study the role of the exposome and novel environmental factors in IBD.

Whereas the role of the genome has been subject to extensive research already, the exact role of the exposome remains unclear. Therefore, future studies should focus on the role of the exposome. In order to acquire generalizable results and enable comparison between different study cohorts, one extensive measurement tool should be used world-wide. The GIEQ will be used in the 1000IBD cohort previously mentioned, providing insight in the role of environmental factors on disease development by evaluation of exposures before diagnosis as well as their role in disease course by evaluation of current exposures. Furthermore, the GIEQ offers the unique opportunity to compare IBD data with 167,000 population-based individuals from the LifeLines study living in the same geographic region. This allows previously identified as well as so far unknown potentially involved environmental factors to be analyzed. The GIEQ provides the opportunity to measure a large amount of environmental exposures all at once, allowing analysis of interactions between different environmental exposures, as different exposures seem to have similar biological modus of effect. Gut permeability for example, seems to be affected by the use of non-steroidal anti-inflammatory drugs (NSAIDs) as well as smoking cigarettes [32, 33]. One can imagine that combining both exposures will yield a different effect than that of individual evaluation per each factor.

As with all questionnaire-based research, the GIEQ has its limitations. Recall bias might lead to wrongful or incomplete answering, with increasing risk for questions concerning the past (e.g., before diagnosis of IBD), especially in older patients. However, since our validation cohort was significantly older, accompanied by an increased disease duration, when compared to the complete IBD cohort, it is conceivable that the results of our validation analyses are accurate, if not an underestimate of actual reliability. As the results have shown, we observed proportional bias for the calculated activity scores. This is likely due to better understanding of the used SQUASH-format in the second fill, leading to better completion and therefore higher activity scores. In future use of the questionnaire, this has to be taken into account. Due to the size of our validation cohort, separate validation for certain subgroups of patients (e.g., men versus women or different age groups) was not feasible. However, there is no evidence that the results of validation might have been different in subgroups than in the whole cohort, and according to found results, the sample size is sufficient. Our strategy to develop the GIEQ was to be as inclusive as possible, in sense of including comprehensive items assessing any known potentially involved environmental factor. Though, this may lead to a longer duration of completing the GIEQ. Patients use approximately an hour to fill the web-based questionnaire, which may lead an unwitting lack of precision and consistency in filling the questionnaire and thus introducing information and attrition bias when generating large study cohorts by using GIEQ. However our validation shows good equality between first and second measurements suggesting consistent answers to questions by patients. Also, to reduce this limitation, the web-based GEIQ was designed with an automatic save function at any given moment. Patients may stop filling the questionnaire for any reason at any time, and proceed at a later time point, without the loss of work. Finally, a Dutch version of the GIEQ was used, due to our Dutch patient cohort. However, an English translation of the GIEQ is readily available and can be validated in and used for studying English speaking IBD cohorts using our validation methods. As previous studies have shown differences in the role and impact of different factors of the exposome between Asian and Western study populations, the need for a universal study method is further clarified. [8, 9, 34] Future studies world-wide using the GIEQ are therefore strongly encouraged.

As past research in the field of genetics has thought us, the exposome should not be studied solitary. To examine IBD etiology as a whole, future studies should be focused on multiple levels of information. Within the UMCG, this view led to a multi-omics approach and the 1000IBD cohort. (Imhann et al., submitted) Due to the study design of the 1000IBD cohort, a prospective cohort in which over a thousand participants are monitored, and data is collected on phenotype, microbiome and genome, addition of the GIEQ allows genome-exposome and microbiome-exposome interaction analysis. Combining all these will not only give new insights into IBD etiology but will also be aimed at discovering biomarker profiles and treatment targets and will form an important step in progression of IBD research. The GIEQ forms an important step towards this goal.

At present, it becomes more evident that while genomic information alone may not explain disease susceptibility and shows little predictability of IBD in the general population, there is an increasing need to comprehensively measure environmental factors in a standardized and harmonized fashion. The GIEQ is among the first feasible tools able to measure an extended number of environmental factors, with a convenient level of reliability and reproducibility, whilst universally applicable to examine the exposome across IBD cohorts. Implementation of the GIEQ offers the chance to collect standardized information concerning the exposome in IBD and opens the possibility to perform large-scale meta exposome association studies to further understand the pathogenesis of IBD and improve the predictability of the occurrence of IBD and its course. Besides using the GIEQ for secondary prevention by identifying patients with a high-risk exposome profile, lessons learned by studying IBD cohorts can be applied to the general population. Primary prevention might become a possibility when persons at risk for disease development can be identified based on a high exposome risk score combined with genetic susceptibility for disease development, as is particularly important in westernizing countries around the world.

## Electronic supplementary material

Below is the link to the electronic supplementary material.

Supplementary material 1 (XLSX 455 kb)

Supplementary material 2 (DOCX 258 kb)

Supplementary material 3 (TIFF 798 kb)

Supplementary material 4 (DOCX 12 kb)
