# Supplementary material for: Development and validation of a web-based questionnaire to identify environmental risk factors for inflammatory bowel disease: the Groningen IBD Environmental Questionnaire (GIEQ)
Source: J Gastroenterol. 2018 Aug 14;54(3):238–48. doi: 10.1007/s00535-018-1501-z (PMC6394725; doi:10.1007/s00535-018-1501-z)
Supplement: Supplementary file 2 — Supplementary material 2 (DOCX 258 kb) [file 535_2018_1501_MOESM2_ESM.docx]

**Online supplementary material 2** – Bland-Altman plots

Category: sleep

*Calculated duration of sleep on workdays, before IBD diagnosis (variable name: “GeslapenUren1t”)*

One sample T-test: mean (SD) = -182.73 (4027.07), p-value = 0.71

Bland-Altman plot:


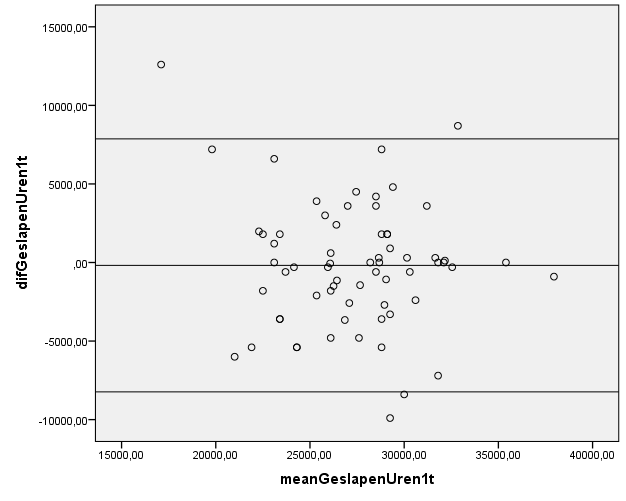


Linear regression: p-value = 0.34

*Calculated duration of sleep on weekend days, before IBD diagnosis (variable name: “GeslapenUren1t”)*

One sample T-test: mean (SD) = 148.18 (4500.39), p-value = 0.79

Bland-Altman plot:


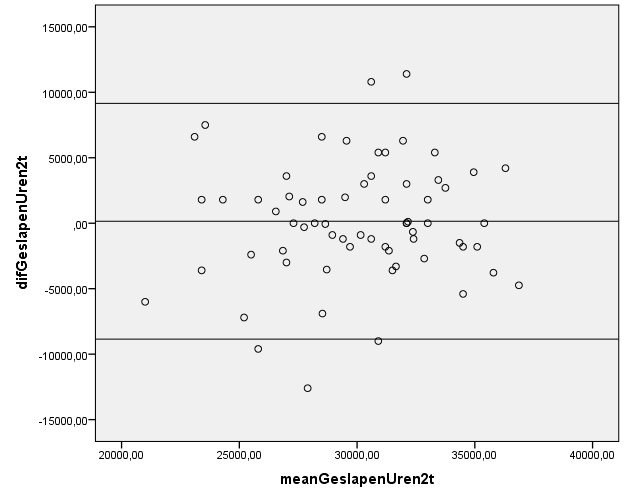


Linear regression: p-value = 0.56

*Patient estimation of average duration of sleep, before IBD diagnosis (variable name: “SLEEP4XTXT”)*

One sample T-test: mean (SD) = -0.0213 (0.794), p-value = 0.85

Bland-Altman plot:


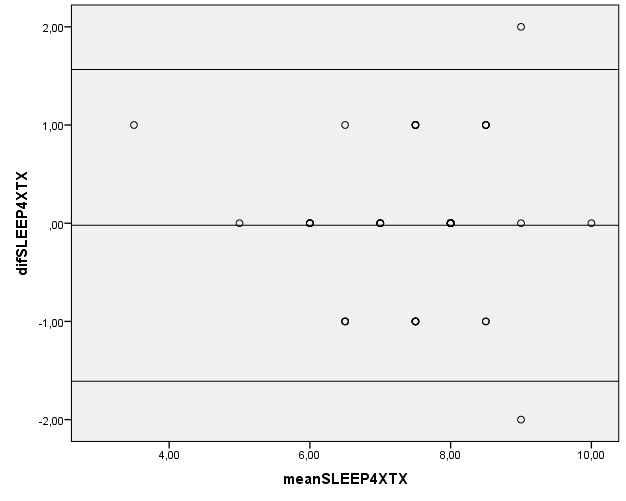


Linear regression: p-value = 1.00

*Calculated duration of sleep on workdays, currently (variable name: “GeslapenUren1Nt”)*

One sample T-test: mean (SD) = -96.0 (3086.46), p-value = 0.80

Bland-Altman plot:


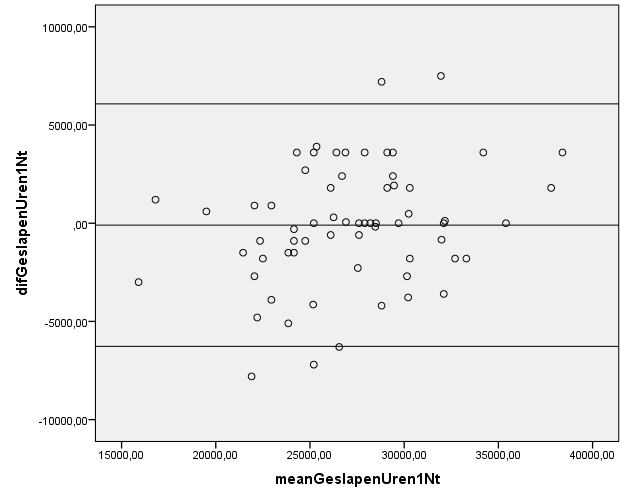


Linear regression: p-value = **0.025**

Linear regression of log transformed values p-value = 0.38

*Calculated duration of sleep on weekend days, current situation (variable name: “GeslapenUren1Nt”)*

One sample T-test: mean (SD) = 38.4 (3590.5), p-value = 0.93

Bland-Altman plot:


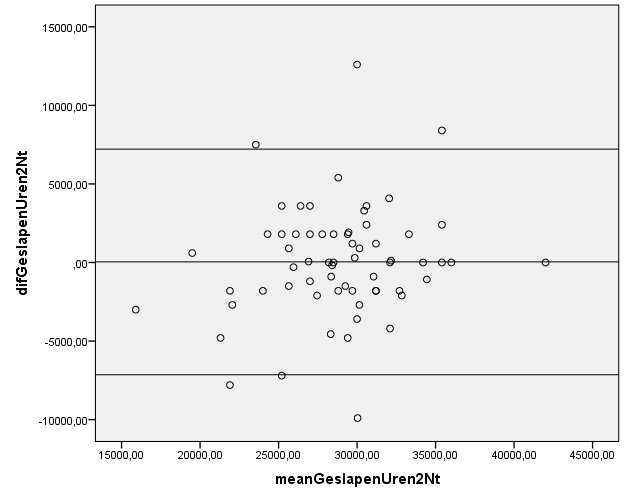


Linear regression: p-value = 0.18

*Patient estimation of average duration of sleep, current situation (variable name: “SLEEP4NXTXT”)*

One sample T-test: mean (SD) = -0.08 (0.69), p-value = 0.36

Bland-Altman plot:


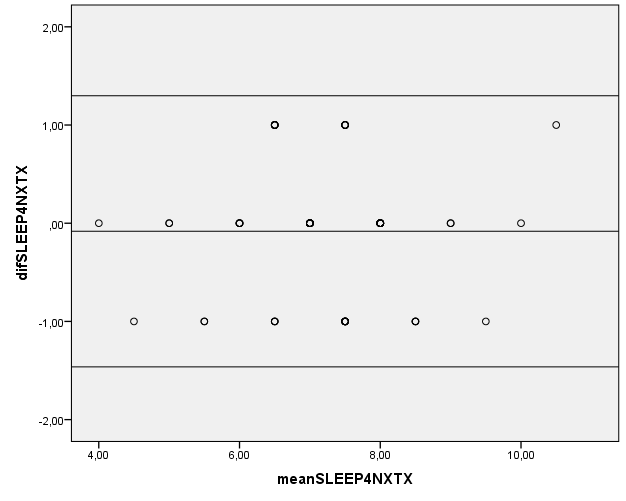


Linear regression: p-value = 0.93

Category: physical activity

*Sum score of physical activity before IBD diagnosis (variable name: “PAtotscr”)*

One sample T-test: mean (SD) = 35.85 (4983.58 ), p-value = 0.95

Bland-Altman plot:


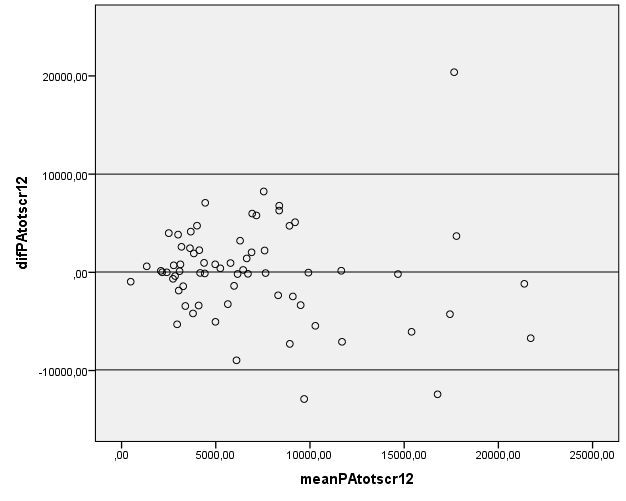


Linear regression: p-value = 0.38

*Sum score of physical activity, current situation (variable name: “NPAtotscr”)*

One sample T-test: mean (SD) = 1004.68 (5196.0), p-value = 0.13

Bland-Altman plot:


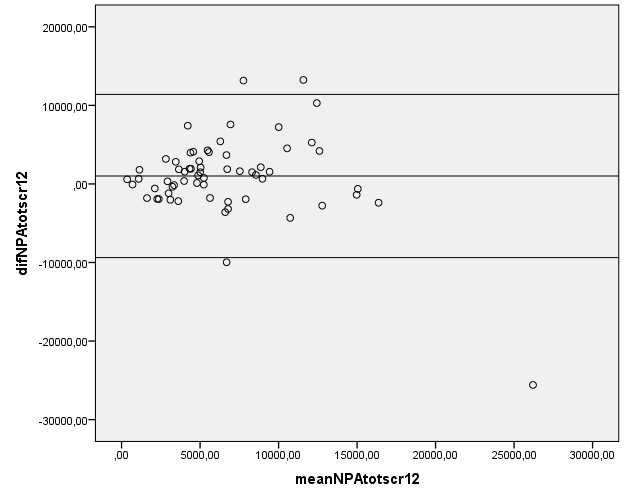


Linear regression: p-value = 0.03

After logarithmic-transformation: linear regression: p-value = 0.032

Category: stress

*Sum score of stressful life-events occurred before IBD diagnosis (variable name: “BRUGHAbefore”)*

One sample T-test: mean (SD) = 0.206 (1.47), p-value = 0.27

Bland-Altman plot:


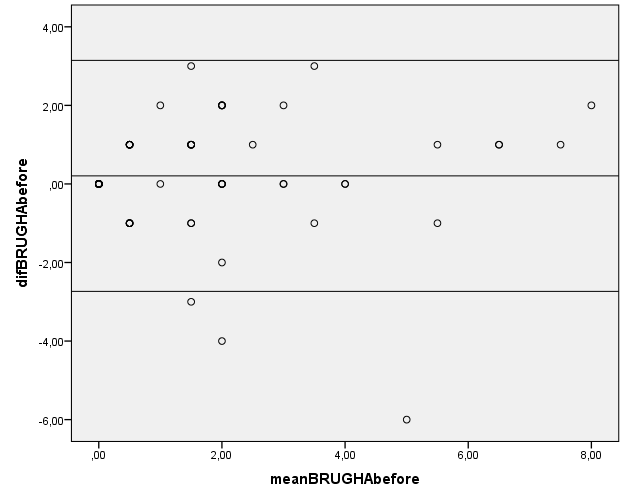


Linear regression: p-value = 0.66

*Sum score of stressful life-events occurred since IBD diagnosis (variable name: “BRUGHAsince”)*

One sample T-test: mean (SD) = -0.06 (1.43), p-value = 0.734

Bland-Altman plot:


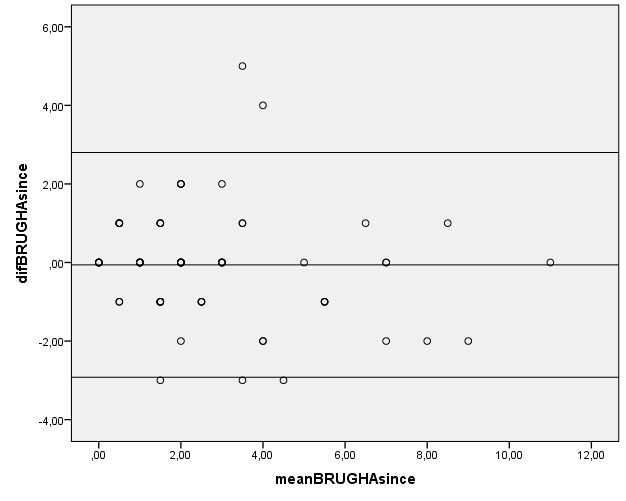


Linear regression: p-value = 0.205

*Sum score of stress in different aspects of life before IBD diagnosis (variable name: “GLLMbefore”)*

One sample T-test: mean (SD) = 0.52 (2.81), p-value = 0.13

Bland-Altman plot:


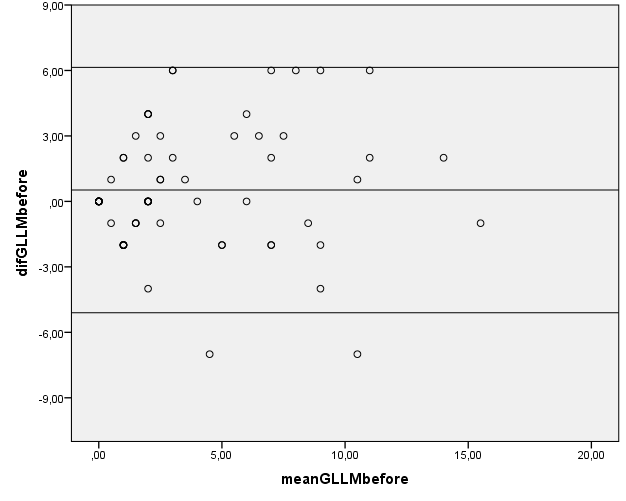


Linear regression: p-value = 0.41

*Sum score of stress in different aspects of life since IBD diagnosis (variable name: “GLLMafter”)*

One sample T-test: mean (SD) = 1.06 (2.58), p-value = 0.001

Bland-Altman plot:


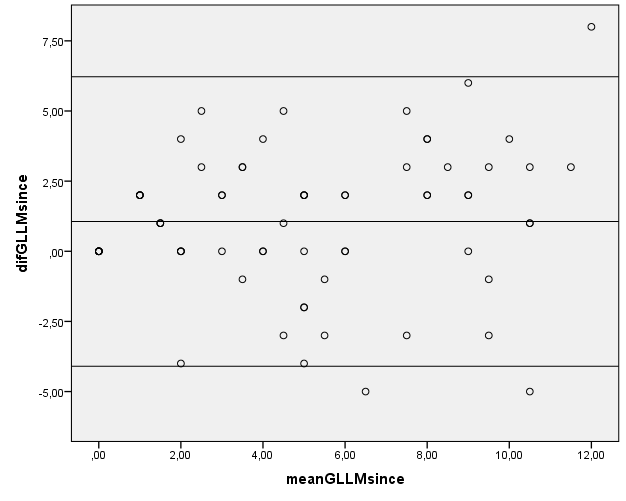


Linear regression: p-value = 0.20

Category: character

*Sum score competence (variable name: “sumC”)*

One sample T-test: mean (SD) = -0.366 (2.95), p-value = 0.43

Bland-Altman plot:


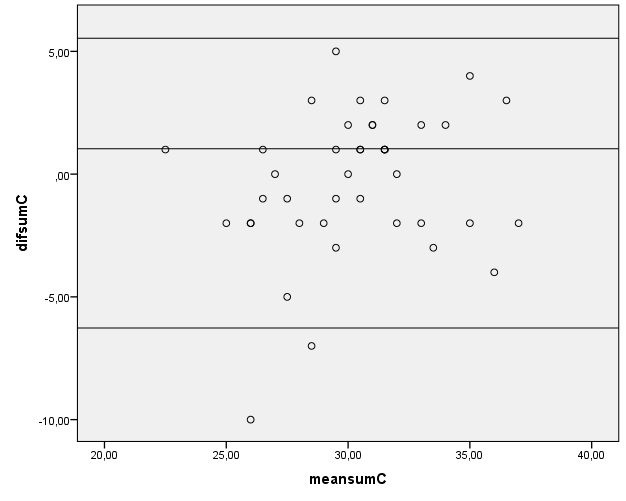


Linear regression: p-value = 0.151

*Sum score anger and hostility (variable name: “sumA”)*

One sample T-test: mean (SD) = 1.046 (3.27), p-value = 0.04

Bland-Altman plot:


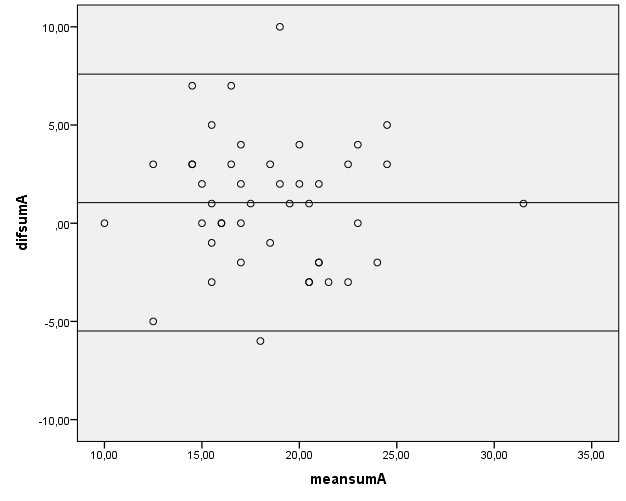


Linear regression: p-value = 0.834

*Sum score self-consciousness (variable name: ”sumSC”)*

One sample T-test: mean (SC) = 0.610 (0.257), p-value = 0.14

Bland-Altman plot:


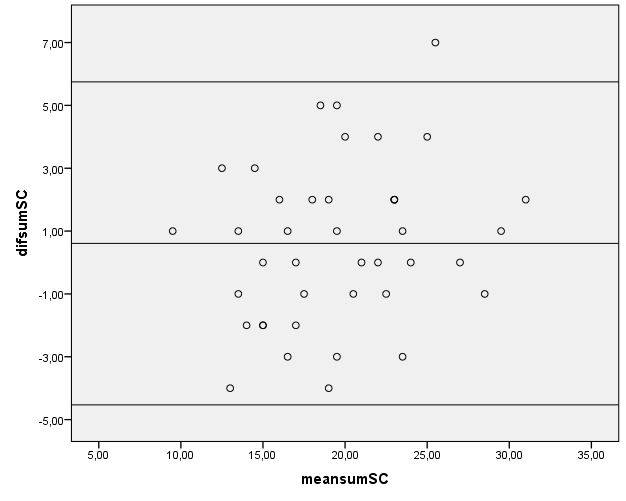


Linear regression: p-value = 0.16

*Sum score impulsivity (variable name: “ sumI”)*

One sample T-test: mean (SD) = 0.308 (3.079), p-value = 0.54

Bland-Altman plot:


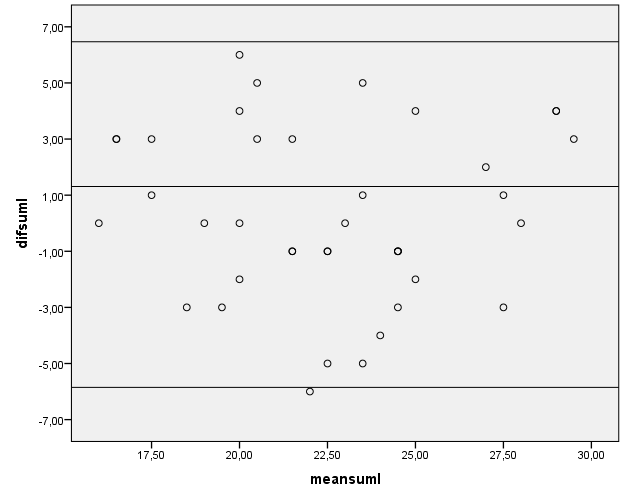


Linear regression: p-value = 0.89

*Sum score excitement (variable name: “sumE”)*

One sample T-test: mean (SD) = 0.524 (2.973), p-value = 0.26

Bland-Altman plot:


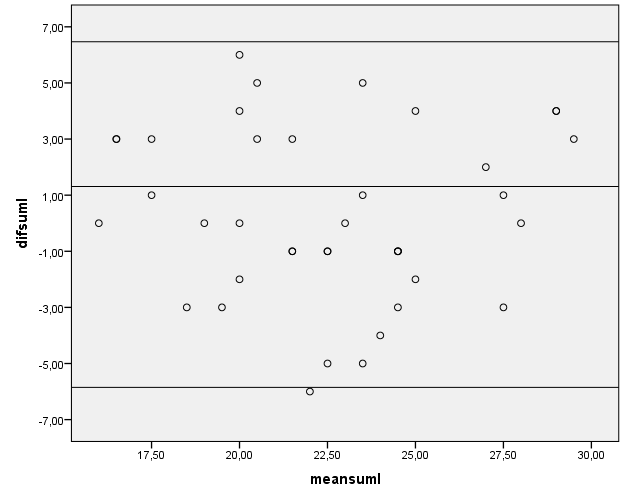


Linear regression: p-value = 0.29

*Sum score self-discipline (variable name: “sumSD”)*

One sample T-test: mean (SD) = -0.1 (2.61), p-value = 0.81

Bland-Altman plot:


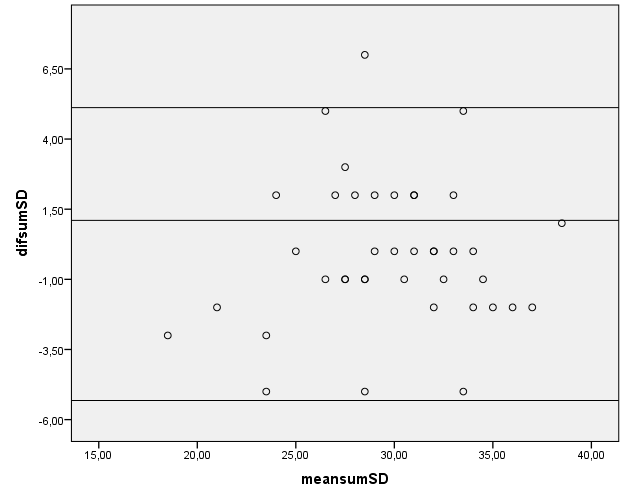


Linear regression: p-value = 0.71

*Sum score vulnerability (variable name: “sumV”)*

One sample T-test: mean (SD) = 0.119 (2.96), p-value = 0.80

Bland-Altman plot:


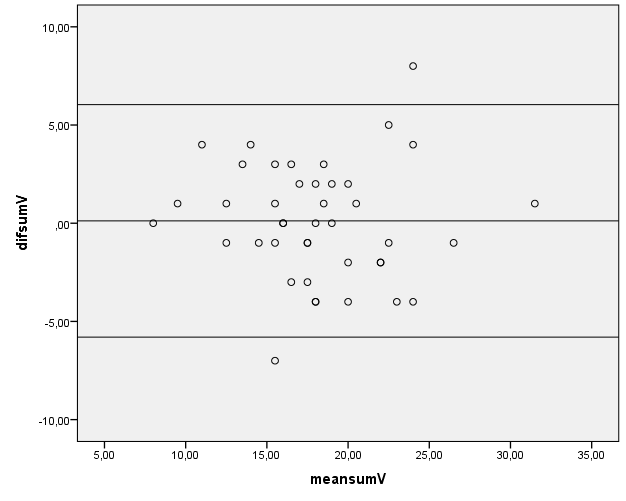


Linear regression: p-value = 0.83

*Sum score deliberation (variable name: “sumD”)*

One sample T-test: mean (SD) = 0.105 (2.66), p-value = 0. 81

Bland-Altman plot:


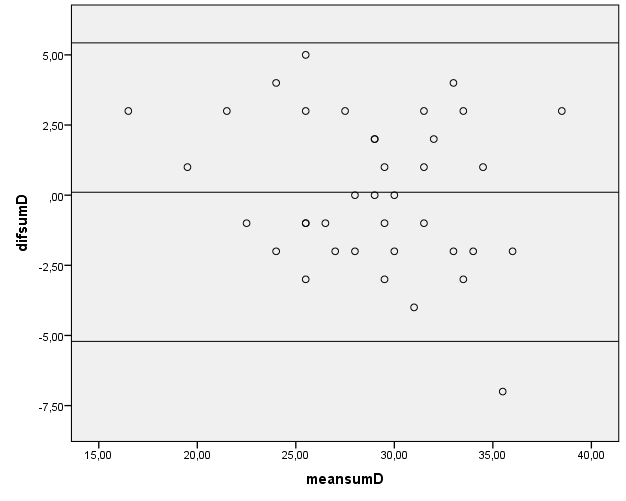


Linear regression: p-value = 0.17

*Sum score total (variable name: “sumTOT”)*

One sample T-test: mean (SD) = 3.10 (8.64), p-value = 0. 12

Bland-Altman plot:


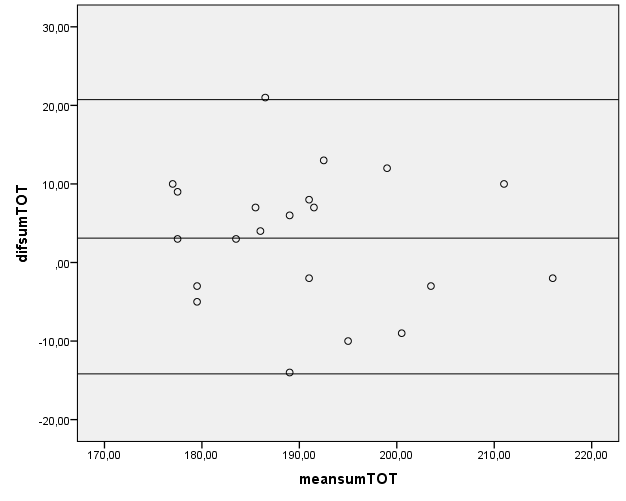


Linear regression: p-value = 0.58
