# Supplementary figures and images for: Development and validation of a web-based questionnaire to identify environmental risk factors for inflammatory bowel disease: the Groningen IBD Environmental Questionnaire (GIEQ)
Source: J Gastroenterol. 2018 Aug 14;54(3):238–48. doi: 10.1007/s00535-018-1501-z (PMC6394725; doi:10.1007/s00535-018-1501-z)

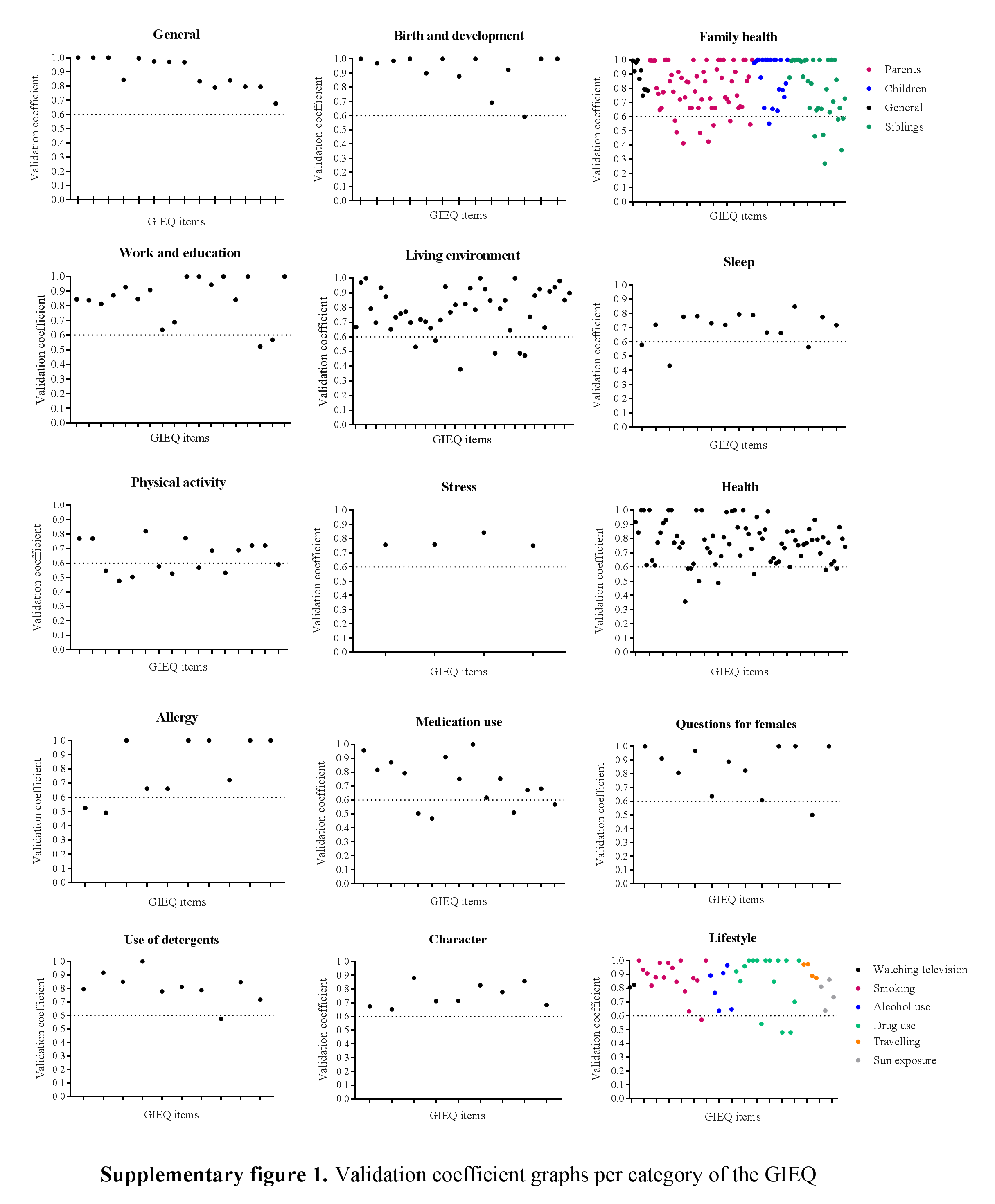

Supplement: Supplementary file 3 — Supplementary material 3 (TIFF 798 kb) [file 535_2018_1501_MOESM3_ESM.tif]
