# Supplementary material for: Development and validation of a web-based questionnaire to identify environmental risk factors for inflammatory bowel disease: the Groningen IBD Environmental Questionnaire (GIEQ)
Source: J Gastroenterol. 2018 Aug 14;54(3):238–48. doi: 10.1007/s00535-018-1501-z (PMC6394725; doi:10.1007/s00535-018-1501-z)
Supplement: Supplementary file 4 — Supplementary material 4 (DOCX 12 kb) [file 535_2018_1501_MOESM4_ESM.docx]

| **Supplementary table 1.** Comparison of GIEQ results to longitudinal prospective 1000IBD cohort | | | | |
| --- | --- | --- | --- | --- |
|  |  | GIEQ | 1000IBD | Validation coefficient |
| Cigarette smoking, ever | *n (%)* | 43 (62.3) | 43 (67.2) | 0.88^a^ |
| Cigarette smoking, current | *n (%)* | 14 (20.3) | 13 (18.8) | 0.90^a^ |
| Passive smoke exposure, childhood | *n (%)* | 50 (72.5) | 42 (70.0) | 0.78^a^ |
| Tonsillectomy, ever | *n (%)* | 35 (55.6) | 32 (52.5) | 0.92^a^ |
| Educational level  Low  High | *n (%)*  *n (%)* | 28 (38.4)  45 (61.6) | 25 (39.7)  38 (60.3) | 0.86^a^ |
| No. of children | *median (IQR)* | 2 (0-2) | 1 (0-2) | 0.95^b^ |
| Family members affected with IBD  Child(ren)  Sibling(s) | *n (%)*  *n (%)* | 2 (2.6)  4 (5.3) | 2 (3.2)  4 (6.5) | 1.0^a^  0.85^a^ |
| ^a^ Kappa coefficient, ^b^ Spearman correlation coefficient | | | | |
